# Quantification of subcortical gray-matter vascularization using 7 Tesla time-of-flight angiography

**DOI:** 10.1002/brb3.154

**Published:** 2013-07-14

**Authors:** Mathias Laurig, Xinyang Liu, Michael Wyss, Anton Gietl, Lena Jellestad, Roger M Nitsch, Klaas Prüssmann, Christoph Hock, Paul G Unschuld

**Affiliations:** 1 Division of Psychiatry Research and Psychogeriatric Medicine, University of ZürichZürich, Switzerland; 2 Brigham and Women's Hospital, Harvard Medical SchoolBoston, Massachusetts; 3 Institute for Biomedical Engineering, Swiss Federal Institute of Technology (ETH Zürich)Zürich, Switzerland

**Keywords:** 7 Tesla, aging, cerebrovascular integrity, high field strength, MRI

## Abstract

**Background** The integrity of subcortical brain nuclei is associated with maintenance of regular cognitive performance levels and has been shown to be particularly affected by aging-related vascular pathology. This study aims to demonstrate applicability of high field strength magnetic resonance angiography at 7 Tesla (7T) for assessment of interindividual variation in subcortical vascularization. **Methods** Two healthy female subjects without known history of cerebrovascular disease or malformation, aged 43 and 86 years, respectively, were administered three-dimensional (3D) high-resolution time-of-flight (TOF) magnetic resonance angiography at 7T. The FreeSurfer software package was used for automated parcellation and assessment of subcortical volumes. For each volume, mean regional intensities were calculated based on the TOF contrast as a quantitative reflection of regional subcortical gray-matter vascularization. **Results** While volumes of the subcortical brain region assessed did not differ significantly (30.2 and 27.8 mL, *P* = 0.78), mean intensities were significantly reduced in the older participant (10%, *P* = 0.004). Mean intensities could be assessed for each participant for 14 subcortical structures, strongest differences were observable for the left and right Thalamus (*T* [left, right] = 3.85, 3.82; *P* [left, right] = 0.002, 0.003). **Conclusions** High-resolution TOF magnetic resonance angiography may be used in combination with automated volume-based parcellation to quantify regional subcortical vascularization and to assess interindividual differences. Additional studies are necessary to assess its potential use in clinical trials on cerebrovascular integrity in a context of aging-related brain change.

## Introduction

Integrity of subcortical gray matter is fundamental for maintenance of higher level cognitive processing capacities and together with Alzheimers disease (AD), vascular alterations are most common causes for aging-related cognitive impairment (Kling et al. 2013). While subcortical small vessel disease in particular has been associated with cognitive impairment and vascular dementia is closely associated with striatal gray-matter pathology (Mori 2002; Roman et al. 2002; Swartz et al. 2008; Scimeca and Badre 2012) postmortem brain analysis suggests a high degree of comorbidity between vascular pathology and neurodegenerative disorders such as AD (Kling et al. 2013).

Three-dimensional (3D) time-of-flight (TOF) magnetic resonance (MR)-angiography utilizes magnetic differences between flowing blood and stationary tissues as a contrast and can be used at high field strengths of 7 Tesla (7T) for assessment of striatal gray-matter vascular integrity (Cho et al. 2008; Hendrikse et al. 2008).

While subcortical gray-matter nuclei show distinct changes in relation to increased age (Murphy et al. 1992; Cherubini et al. 2009; Long et al. 2012), to our knowledge no studies have been performed using TOF angiography to assess and quantify this otherwise well documented relationship. We therefore used high-resolution 3D-TOF MR-angiography at high field strength of 7T to assess individual vascularization of subcortical gray-matter nuclei in relation to age. Subcortical vascularization was assessed as a quantitative trait based on the regional TOF contrast defined by the volume-based subcortical parcellation algorithm included in the FreeSurfer software package.

## Methods

Two healthy female volunteers (subject #1, aged 43 and subject #2, 86 years) were recruited through the division of Psychogeriatric Medicine, University of Zürich and signed informed consent. Both received a physical and psychiatric examination to exclude manifest medical or neuropsychiatric disorder and scored 30 of 30 points on the Mini-mental state test, indicating normal cognitive performance levels (Folstein et al. 1975).

TOF MR-angiography was performed on a 7.0-Tesla Philips Achieva high-field MR system (Philips Healthcare, Cleveland, OH) using a quadrature transmit head coil together with a 16-channel receive array (NOVA Medical, Wilmington, NC). A 3D gradient echo sequence was used with 0.24 × 0.4 mm inplane resolution, field of view (FOV) 200 × 190 mm, echo time (TE) 3.4 msec, Repetition Time (TR) 20 msec, Flip angle 20°, 300 slices, thickness 0.6 mm in transversal orientation. SENSE was applied in the right–left direction with a SENSE factor of 3. The images were reconstructed to a Voxel size of 0.25 × 0.25 × 0.3 mm. The scan duration was ∼11 min and 43 sec. Targeted maximum intensity projection was made for the region of interest, which focused onto the main trunk of the middle cerebral artery and the anterior cerebral artery.

Volumetric segmentation was performed with the Freesurfer image analysis suite, which is documented and freely available for download online (http://surfer.nmr.mgh.harvard.edu/). Briefly, this processing includes removal of nonbrain tissue using a hybrid watershed/surface deformation procedure, automated Talairach transformation, segmentation of the subcortical white-matter and deep gray-matter volumetric structures, and intensity normalization for the TOF contrast. Mean intensities and standard deviations were calculated for each volume in both participants and used for nonparametric testing (Wilcoxon rank-sum test) followed by a correction for multiple comparisons (Holm 1979), adjusting *P*-values for testing multiple hypotheses on effects pertaining to the 14 selected subcortical structures.

## Results

**Figure 1:**
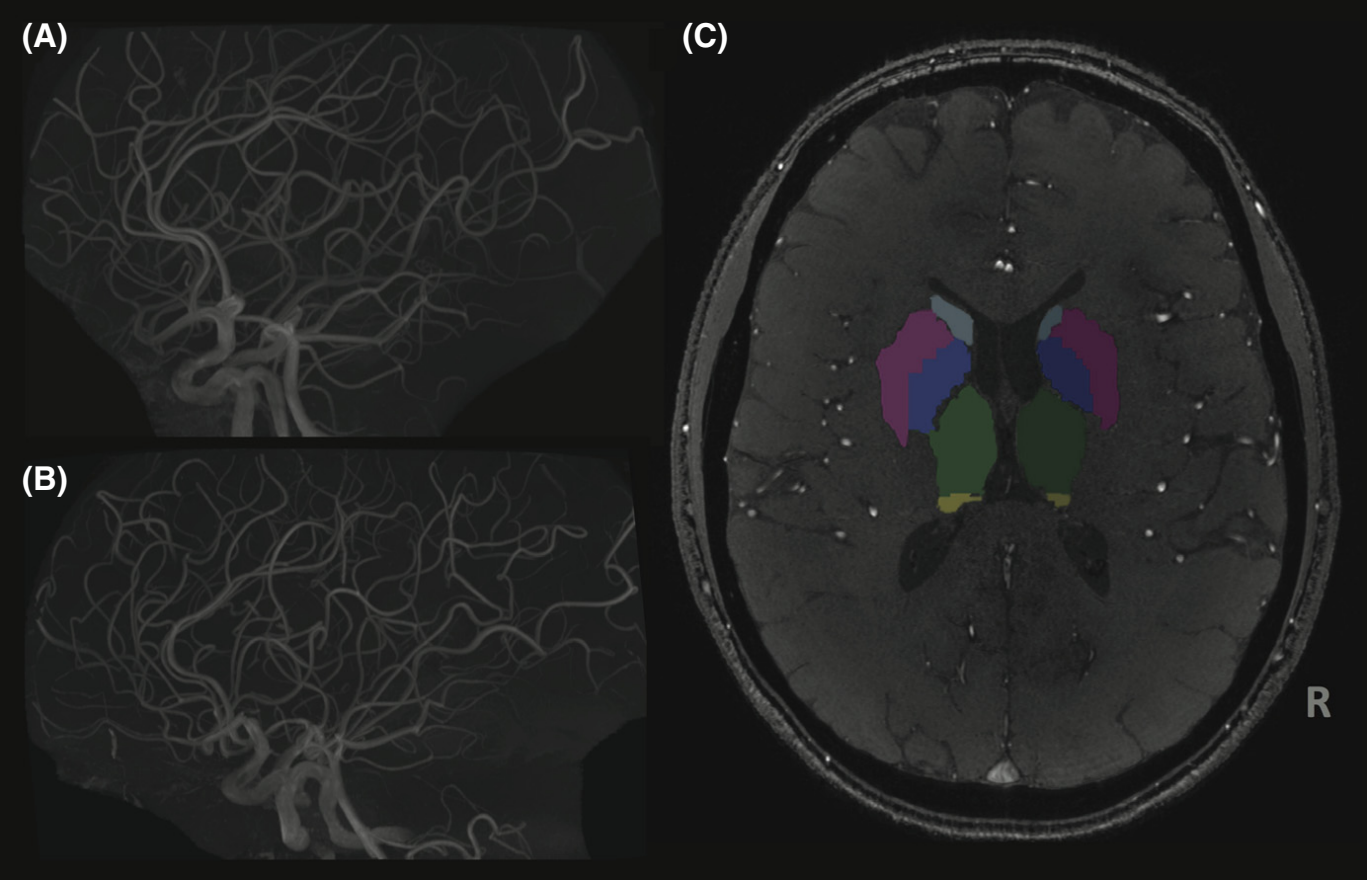
(A and B) Reconstructed three-dimensional (3D) time-of-flight (TOF) images, demonstrating subcortical and cortical vessels originating from the main trunks of the cerebral arteries for both subjects assessed. (C) Indicates the subcortical brain areas assessed by FreeSurfer and tested for mean intensities derived from the TOF contrast. (“R” indicates right side).

**Figure 2:**
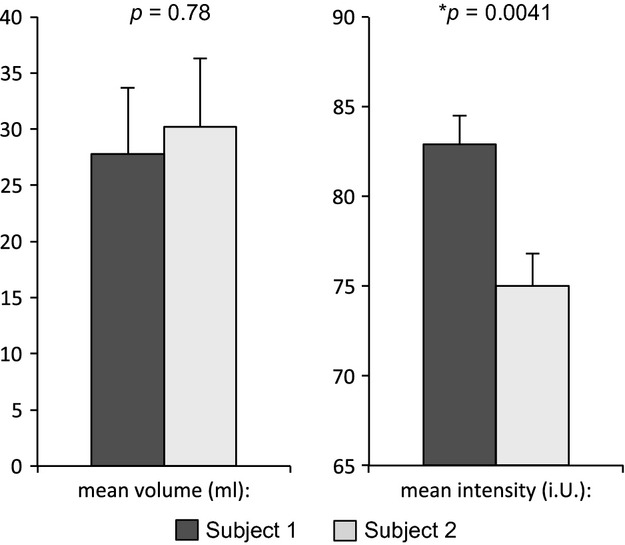
Mean values (SEM) of total subcortical gray-matter intensity and total volume of the subcortical gray-matter structures assessed for subject #1 and #2.

For volumetry and assessment of quantitative correlates of vascularization, subcortical segmentation of gradient echo sequences and the respective TOF MR-angiography volumes was performed using the FreeSurfer image analysis suite (Fig. 1). Assessment of whole-brain TOF contrast indicated significantly lower intensity values in subject #2 versus subject #1 for the Thalamus (left: −9.9%, *P* = 0.002, right: −10.0%, *P* = 0.003), right Caudate (−8.3%, *P* = 0.044), and Pallidum (left: −17.3%, *P* = 0.011, right: −13.1%, *P* = 0.02). No significant differences in intensity were observed for the left Caudate (*P* = 0.07), Putamen (left: *P* = 1, right: *P* = 0.474), Hippocampus (left: *P* = 1, right: *P* = 1), Amygdala (left: *P* = 1, right: *P* = 1), and the Accumbens-area (left: *P* = 1, right: *P* = 1). Also mean intensity of all 14 structures was significantly lower in subject #2 than in subject #1 (−10%, means [SEM] subject #1: 82.9 [1.6]; subject #2: 75.0 [1.8]; *P* = 0.004). There was no significant difference between both subjects observable regarding total volume of the 14 subcortical gray-matter structures assessed (means [SEM] subject #1: 30.2 mL [6.1]; subject #2: 27.8 mL [5.9]; *P* = 0.078) (Table 1 and Fig. 2).

**Table 1:** Subcortical regions identified by the parcellation algorithm and estimated volumes for each participant, as well as differences in mean regional intensity and the respective *T*-test statistics

	Volume (mL)			*T*-test	
			
Structure	Subject 1	Subject 2	*D* mean intensity (%) Subject 2–Subject 1	*T*	*P* (adjusted)
Thalamus (L.)	77.2	77.0	−9.9	−3.85	*0.002
Thalamus (R.)	64.8	70.0	−10.0	−3.82	*0.003
Caudate (L.)	15.8	29.7	−10.3	−2.96	0.07
Caudate (R.)	29.1	44.5	−8.3	−3.06	*0.044
Putamen (L.)	41.0	48.3	−4.2	−1.78	1
Putamen (R.)	39.9	33.8	−4.6	−2.16	0.474
Pallidum (L.)	17.2	22.7	−17.3	−3.65	*0.011
Pallidum (R.)	23.1	19.9	−13.1	−3.43	*0.02
Hippocampus (L.)	32.3	22.2	−2.0	−0.26	1
Hippocampus (R.)	24.3	28.6	−9.5	−1.49	1
Amygdala (L.)	7.8	9.0	−11.7	−1.14	1
Amygdala (R.)	9.8	7.7	5.0	0.98	1
Accumbens-area (L.)	3.5	5.7	−6.5	−0.53	1
Accumbens-area (R.)	3.4	3.9	−28.2	−1.7	1

*P-values are corrected for multiple testing, significant differences are indicated in bold.

## Discussion

In this study, we quantified individual aging-related decrease of subcortical gray-matter vascularization and demonstrated most pronounced changes for brain regions in the Thalamus and Pallidum. By using 3D-TOF angiography at high field strength of 7T, high spatial resolution could be realized, allowing to take into account potential regional small vessel pathology.

While our findings need to be interpreted with caution as only two subjects were included in the current study, they nevertheless appear consistent with earlier reports on subcortical alterations in the context of aging that also point toward particular vulnerability of subcortical gray-matter nuclei including particularly thalamic brain regions (Murphy et al. 1992; Cherubini et al. 2009; Long et al. 2012). Furthermore, as we find a reduction of vascular signal in striatal gray matter but no significant difference in total volume, this may also support earlier considerations on a prominent role of vascular pathology in the process of aging-related changes of striatal gray matter (Mori 2002; Roman et al. 2002; Kling et al. 2013), observable on a single subject level.

To our knowledge, this is the first study to use magnetic resonance imaging (MRI)-angiography for assessment of aging-related subcortical gray-matter vascularization and also the first to use TOF-MRI at 7T in combination with an automated parcellation algorithm to assess quantifiable indicators of subcortical vascular integrity.

TOF-MRI is routinely used for assessment of cerebral vascular pathology and related subcortical gray-matter integrity. Using higher field strength in MRI applications is associated with significantly increased Signal to noise ratio (SNR) (Pruessmann 2004; Lu et al. 2005) and performing MRI-TOF angiography at 7T has been demonstrated to make possible the high spatial resolutions necessary for the assessment of small subcortical vessels, which have been shown to be particularly vulnerable in the process of aging (Cho et al. 2008; Hendrikse et al. 2008; Madai et al. 2012). It has to be taken into account, however, that regional inhomogeneities due to the high fieldstrength at 7T may result in inconsistencies of the effective flip-angel in TOF-MRI (Pruessmann 2004). To minimize this issue, maximum intensity projection was focused on the subcortical region of interest.

Taken together, our study demonstrates interindividual differences in subcortical vascularization that possibly reflect aging-related vulnerability of gray-matter nuclei for vascular pathology (Murphy et al. 1992; Cherubini et al. 2009; Long et al. 2012). While we find most prominent changes for the thalamic region, our data may reflect reduced vascular activity as a proxy of reduced gray-matter viability (Kling et al. 2013). Moreover, our data demonstrate the applicability of TOF angiography together with the FreeSurfer subcortical parcellation algorithm on the single subject level, resulting in a quantifiable measure of regional subcortical vascularization. Additional studies are needed to validate this approach and to determine applicability as an outcome marker in therapeutic trials focussed on vascular integrity in the context of aging-related neuropsychiatric disorder.
